# Point-dipole approximation for small systems of strongly coupled radiating nanorods

**DOI:** 10.1038/s41598-019-41327-6

**Published:** 2019-04-05

**Authors:** Derek W. Watson, Stewart D. Jenkins, Vassili A. Fedotov, Janne Ruostekoski

**Affiliations:** 1 Mathematical Sciences and Centre for Photonic Metamaterials, University of Southampton, Southampton, SO17 1BJ United Kingdom; 2 Optoelectronics Research Centre and Centre for Photonic Metamaterials, University of Southampton, Southampton, SO17 1BJ United Kingdom; 3 Department of Physics, Lancaster University, Lancaster, LA1 4YB United Kingdom

## Abstract

Systems of closely-spaced resonators can be strongly coupled by interactions mediated by scattered electromagnetic fields. In large systems the resulting response has been shown to be more sensitive to these collective interactions than to the detailed structure of individual resonators. Attempts to describe such systems have resulted in point-dipole approximations to resonators that are computationally efficient for large resonator ensembles. Here we provide a detailed study for the validity of point dipole approximations in small systems of strongly coupled plasmonic nanorods, including the cases of both super-radiant and subradiant excitations, where the characteristics of the excitation depends on the spatial separation between the nanorods. We show that over an appreciable range of rod lengths centered on 210 nm, when the relative separation *kl* in terms of the resonance wave number of light *k* satisfies $kl\gtrsim \pi /2$, the point electric dipole model becomes accurate. However, when the resonators are closer, the finite-size and geometry of the resonators modifies the excitation modes, in particular the cooperative mode line shifts of the point dipole approximation begin to rapidly diverge at small separations. We also construct simplified effective models by describing a pair of nanorods as a single effective metamolecule.

## Introduction

Plasmonic nanorods are the most basic form of optical resonators. The scattering of light from any resonator has the ability to produce strong interactions that can result from the wave repeatedly scattering off the same resonator. The EM coupling between different resonators results in different eigenmodes of response, the strong interaction limits of which can be most easily achieved in microwave systems with low ohmic losses, but can also manifest in plasmonic systems^1,2^. The eigenmodes can also destructively interfere and manifest as Fano resonances in the transmitted field^3–5^, whose narrow resonances potentially make them useful in applications such as plasmonic rulers^6^ and biosensors^7^. Designing material structures to support Fano resonances is difficult; not least due to the complex interactions of different modes, but variations in the resonators can affect the line shifts and widths of the interacting modes also^8^.

Apart from applications in plasmonics and nanophotonics, optical resonators (and nanorods in particular) that are much smaller than the wavelength of the driving light are now commonly used as the building blocks of metamaterials - artificial material composites that are designed to interact with light in ways no conventional materials can. Functionalities of metamaterials include perfect absorption^9^ and optical magnetism^10^; with potential applications ranging from cloaking^11–13^ to perfect lenses^14–16^. An important part of understanding how metamaterials realize their functions is knowledge of the electromagnetic (EM) fields scattered by the constituent resonators.

Metamaterials that exhibit strong collective interactions are becoming increasingly popular with experimentalist^2,17–23^. However, modeling the EM interactions in large resonator systems is generally challenging. The interactions among the resonators can be simplified, e.g., by treating the array as an infinite lattice^24,25^ or the resonators as point multipole sources^2,26–28^. Point multipole descriptions in particular have been successful in modeling the cooperative response in planar arrays, e.g., developing electron-beam-driven light sources^29^ and transmission properties^30,31^. Point dipole descriptions can also be extended to more complex metamolecules, such as those exhibiting toroidal dipoles^32^.

Here, we analyze the accuracy of the dipole approximation in more detail in small systems of plasmonic nanorods and show, qualitatively, at what separations the finite-size of a nanorod, and its near fields, must be accounted for. Our theoretical model does not require solving the full Maxwell’s equations for a resonator ensemble; which is computationally demanding for more than a few resonators^33^. Rather, we utilize the formalism developed in ref.^27^ to produce a system of coupled equations for the dynamics of the EM interactions of the scattered and incident EM fields. The method relies on capturing the fundamental physics of each resonator, e.g., its decay rate and resonance frequency, relevant for the radiative coupling between resonators. Our model is readily applied to more complex systems such as those whose resonators are distributed over two planes (one above another) with non-uniform orientations, e.g., a toroidal metamolecule^32^.

Finally, in our paper we also provide an alternative approach for treating each element of a nanorod as a separate meta-atom when we model closely spaced nanorods as a single effective metamolecule. This can notably reduce the number of degrees of freedom in the system.

## Method

In analyzing the EM interactions between plasmonic nanorods and an incident EM field, we utilize the general theory derived in detail in ref.^27^, specifically, for the point electric dipole approximation. We regard nanorods as cylinder-shaped resonators and study their longitudinal polarization excitation; where the charge and current oscillations are assumed to be linear along the axis of the nanorod. Here, we provide a brief overview of our model, a more detailed description is provided in the Supplementary Material.

An incident electric displacement field **D**_in_(**r**,*t*) = *D*_in_**ê**_in_exp(*i***k**_in_⋅**r** − *i*Ω_0_*t*) and magnetic induction ${{\bf{B}}}_{{\rm{in}}}({\bf{r}},t)=\sqrt{{\mu }_{0}/{\varepsilon }_{0}}{\hat{{\bf{k}}}}_{{\rm{in}}}\times {{\bf{E}}}_{{\rm{in}}}({\bf{r}},t)$, with frequency Ω_0_, polarization vector **ê**_in_ and propagation vector **k**_in_ drive each resonator *j*’s internal charge and current sources. These source oscillations behave in a manner comparable to an LC circuit with resonance frequency *ω*_*j*_^27^,

1 $${\omega }_{j}=\frac{1}{\sqrt{{L}_{j}{C}_{j}}}\mathrm{.}$$

Here, *L*_*j*_ and *C*_*j*_ are respectively, an effective self-inductance and self-capacitance. A dynamic variable with units of charge *Q*_*j*_(*t*) and its time derivative, the current ${I}_{j}(t)={\dot{Q}}_{j}(t)$, describes the state of current oscillations within each resonator *j*. In order to analyze the coupled equations for the EM fields, we introduce the slowly varying normal mode oscillator amplitudes *b*_*j*_(*t*)^27^, with generalized coordinate the charge *Q*_*j*_(*t*) and conjugate momentum *ϕ*_*j*_(*t*), where

2 $${b}_{j}(t)=\frac{1}{\sqrt{2{\omega }_{j}}}[\frac{{Q}_{j}(t)}{\sqrt{{C}_{j}}}+i\frac{{\varphi }_{j}(t)}{\sqrt{{L}_{j}}}].$$

In the rotating wave approximation the conjugate momentum and current are linearly-proportional^27^. We use Eq. (2) to describe a general resonator with sources of both polarization **P**_*j*_(**r**, *t*) and magnetization **M**_*j*_(**r**,*t*). The resonator’s scattered EM fields result from the oscillations of *Q*_*j*_(*t*) and *I*_*j*_(*t*); which are proportional to **P**_*j*_(**r**, *t*) and **M**_*j*_(**r**, *t*), respectively^27^.

The collective interactions of *N* resonators with each other and an external field is described by the linear system of equations^27^

3 $$\dot{{\bf{b}}}={\mathscr{C}}{\bf{b}}+{{\bf{F}}}_{{\rm{i}}{\rm{n}}}.$$

Here, $\dot{{\bf{b}}}$ is the rate of change of **b**; a vector of *N* normal oscillator variables, and **F**_in_ is a vector describing the interaction of resonator *j* with the incident EM field. The *N*×*N* interaction matrix ${\mathscr{C}}$ requires solving the scattered EM fields for the polarization and magnetization sources. The EM interactions between different resonators *i* ≠ *j* are described by the off-diagonal elements of ${\mathscr{C}}$. The strength of ${{\mathscr{C}}}_{i\ne j}$ depends on the separation and orientation of the resonators, and naturally accounts for the reduced coupling between elements at the edges compared to the center of a lattice, see Supplementary Material. The diagonal elements describe the EM interactions of a resonator with itself, resulting in the resonator’s decay rate Γ_*j*_ and resonance frequency;

4 $${[\begin{array}{c}{\mathscr{C}}\end{array}]}_{jj}=-\frac{{{\rm{\Gamma }}}_{j}}{2}-i({\omega }_{j}-{{\rm{\Omega }}}_{0}\mathrm{).}$$

In our model we consider magnetization due to induced macroscopic currents. In a straight rod even though the induced current is non-zero, it is linear and therefore the corresponding magnetization is negligible; **M**_*j*_(**r**, *t*) ≃ 0. Thus, the scattered EM fields result from a nanorod’s polarization sources **P**_*j*_(**r**, *t*) alone. This results in an effective accumulation of charge on the nanorod’s ends. Analogous simulation methods can also be used to study collective responses of arrays of other resonant emitters, such as atoms^34^.

### Finite-size resonator model

**Figure 1:**
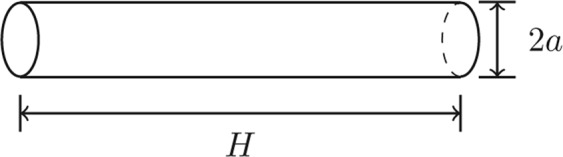
Schematic of a nanorod with radius *a* and length *H*.

For our finite-size model we consider a nanorod with a radius *a* and height *H*_*j*_, see Fig. 1. The polarization density is a uniform distribution of atomic point electric dipoles (with orientation vectors ${\hat{{\bf{d}}}}_{j}$) throughout the volume of the nanorod. For a single nanorod centered at the origin and aligned along the *z* axis, i.e., $\hat{{\bf{d}}}=\hat{{\bf{z}}}$, the spatial profile distribution of the polarization density is

5 $${{\bf{P}}}_{j}({\bf{r}},t)=\frac{{Q}_{j}(t)}{\pi {a}^{2}}\hat{{\bf{z}}}\,{\rm{\Theta }}\,(a-\rho )\,{\rm{\Theta }}\,({H}_{j}/2-z)\,{\rm{\Theta }}\,({H}_{j}/2+z),$$

where Θ is the Heaviside function and *ρ* < *a*. An individual nanorod experiences radiative decay Γ_E1,*j*_, resulting from the interaction of the scattered field with the rod itself. The total decay rate of the rod Γ_*j*_ also includes a phenomenological ohmic loss rate Γ_O,*j*_, where

6 $${{\rm{\Gamma }}}_{j}={{\rm{\Gamma }}}_{{\rm{E1}},j}+{{\rm{\Gamma }}}_{{\rm{O}},j},\,{{\rm{\Gamma }}}_{{\rm{E1}},j}=\frac{{C}_{j}{H}_{j}^{2}{\omega }_{j}^{4}}{6\pi {\varepsilon }_{0}{c}^{3}}.$$

### Point electric dipole approximation

In a point multipole approximation where the length is much less than the wavelength of the incident light $({H}_{j}\ll {\lambda }_{0})$, the nanorod may be approximated as a point electric dipole with polarization density

7 $${{\bf{P}}}_{j}({\bf{r}},t)={Q}_{j}(t){H}_{j}{\hat{{\bf{d}}}}_{j}{\delta }({\bf{r}}-{{\bf{r}}}_{j}\mathrm{).}$$

The orientation of the electric dipole is described by the unit vector ${\hat{{\bf{d}}}}_{j}$ and *H*_*j*_ has dimensions of length. The rate at which the electric dipole radiates as a result of its self interactions is Γ_E1,*j*_. The radiative decay, ohmic losses and total decay rate are given in Equation (6).

### A finite-size effective metamolecule

Thus far, we have only considered the interactions between individual nanorods and point electric dipole resonators. When there are a large number of resonators, one may look to optimize the model. Because resonators are often arranged in a lattice framework, it is possible to consider closely spaced parallel pairs of nanorods as a single effective metamolecule. Here, we extend our finite-size nanorod model to include this effective metamolecule with the same properties as its constituent nanorods, i.e., electric dipole properties.

We consider two parallel nanorods with location vectors ${{\bf{r}}}_{\pm ,j}=[{x}_{j},{y}_{j}\pm l,{z}_{j}]$, and polarization densities **P**_±,*j*_(**r**, *t*), respectively. When *l* is small, we may approximate the pair as a single metamolecule with location vector **r**_*j*_ = [*x*_*j*_, *y*_*j*_, *z*_*j*_]. The metamolecule may be symmetrically excited, i.e., ${{\bf{P}}}_{+,j}({\rm{r}},t)={{\bf{P}}}_{-,j}({\bf{r}},t)$ or it may be antisymmetrically excited, i.e., **P**_+,*j*_(**r**, *t*) = −**P**_−,*j*_(**r**, *t*), where **P**_+,*j*_(**r**, *t*) is defined in Equation (5).

The resonance frequency and radiative decay rate of our effective metamolecule depend on the interactions of the individual nanorods. We calculate these properties later; by analyzing in detail a pair of parallel nanorods and point electric dipoles. In principle, if the nanorods are approximated as point electric dipoles, one may treat a closely spaced parallel pair as a single emitter. If both the electric dipoles are symmetrically excited, there is an effective single point electric dipole^35^. However, if both the dipoles are antisymmetrically excited, there is an effective emitter with both an electric quadrupole and a magnetic dipole moment^35^. In this work, however, we only consider the finite-size effective resonators.

## Results

In this section, we analyze the EM interactions of nanorods, both as point electric dipole emitters and accounting for their finite-size and geometry. We formulate the model for the nanorods by assuming all the nanorods are made of gold and have equal length, i.e., *H*_*j*_ = *H*_0_ for all *j*. As the nanorods are identical, they experience identical ohmic losses, radiative, and total decay rates, i.e., Γ_O,*j*_ = Γ_O_, Γ_E1,*j*_ = Γ_E1_, and Γ_*j*_ = Γ. We choose ${H}_{0}=1.5{\lambda }_{{\rm{p}}}\simeq 210\,{\rm{nm}}$ and radius $a={\lambda }_{p}/5\simeq 28\,{\rm{nm}}$, where ${\lambda }_{{\rm{p}}}\simeq 140\,{\rm{nm}}$ is the plasma wavelength of gold^36^. This yields ${H}_{0}\simeq 0.24{\lambda }_{0}$ and $a\simeq 0.032{\lambda }_{0}$, where ${\lambda }_{0}=2\pi c/{\omega }_{0}\simeq 860\,{\rm{nm}}$ is the (longitudinal) resonance wavelength of the nanorod. Each individual nanorod has a total decay rate Γ, with resulting radiative emission rate ${{\rm{\Gamma }}}_{{\rm{E1}}}\simeq 0.83{\rm{\Gamma }}$ and ohmic loss rate ${{\rm{\Gamma }}}_{{\rm{O}}}\simeq 0.17{\rm{\Gamma }}$. We calculate these parameters in the Supplementary Material, where we employ formulas developed in ref.^37^ for the resonant scattering of light from plasmonic nanoparticles, and ohmic losses are accounted for in the Drude model. To simplify the comparison between our point dipole approximation and finite-size nanorod model, we use the same resonance frequency, radiative decay, ohmic losses and total decay rate in both models.

We analyze the characteristic response of the system in the absence of an incident EM field, studying the characteristic collective modes represented by the eigenvectors **v**_*n*_ of the interaction matrix ${\mathscr{C}}$. The corresponding complex eigenvalues *ξ*_*n*_, describe the collective mode’s characteristic linewidth (real part) and resonance frequency shift (imaginary part); *ξ*_*n*_ = −*γ*_*n*_/2 − *i*(Ω_*n*_ − ω_0_). The number of modes is determined by the number of resonators, and the radiation may be suppressed *γ*_*n*_ < Γ (subradiant), or enhanced *γ*_*n*_ > Γ (superradiant).

### Two parallel nanorods

As our first example, we consider two parallel nanorods (and two point electric dipoles) with location vectors **r**_1_ = −**r**_2_ = [0, *l*/2, 0]. The coupling matrix ${\mathscr{C}}$ has two eigenmodes of current oscillation. In the first mode, the current oscillations are in-phase (symmetric) with ${\hat{{\bf{d}}}}_{1}={\hat{{\bf{d}}}}_{2}$. In the second mode, the current oscillations are out-of-phase (antisymmetric) with ${\hat{{\bf{d}}}}_{1}=-{\hat{{\bf{d}}}}_{2}$. The former we denote by a subscript ‘s’; and latter by a subscript ‘a’.

**Figure 2:**
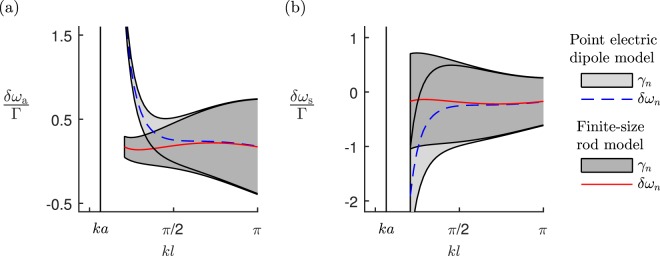
The radiative resonance line shifts *δω*_*n*_ = −(Ω_*n*_ − ω_0_), and linewidths *γ*_*n*_, for the collective out-of-phase (*a*) and in-phase (*b*) eigenmodes, for two parallel nanorods and two parallel point electric dipoles as a function of their separation *l*. The finite-size rods have lengths $H\simeq 0.24{\lambda }_{0}$ and radii $a\simeq 0.032{\lambda }_{0}$. The radiative decay rate of each nanorod is ${\Gamma }_{{\rm{E1}}}\simeq 0.83{\rm{\Gamma }}$, the ohmic losses are ${{\rm{\Gamma }}}_{{\rm{O}}}\simeq 0.17{\rm{\Gamma }}$.

In Fig. 2, we show the collective eigenmode’s radiative resonance line shifts and linewidths for two finite-size parallel nanorods and two parallel point electric dipoles. Throughout the range of *kl*, the decay rates *γ*_*n*_ of the point dipole model closely agree with the corresponding decay rates of the finite-size nanorod model. When *kl* ≈ *π*, the symmetric mode is subradiant with *γ*_s_ ≈ 0.9Γ, and the antisymmetric mode is superradiant with *γ*_a_ ≈ 1.1Γ; for both the nanorods and point electric dipoles. As the separation reduces, the symmetric mode becomes superradiant and the antisymmetric mode subradiant. When *kl* ≈ 2*π*/3, the decay rates of the point multipole approximation are: *γ*_a_ ≈ 0.7Γ (subradiant); and *γ*_s_ ≈ 1.3Γ (superradiant); the finite-size model shows decay rates: *γ*_a_ ≈ 0.8Γ; and *γ*_s_ ≈ 1.2Γ. As the separation becomes small *kl* ≈ π/6, the antisymmetric linewidths approach the ohmic loss rate, *γ*_a_ ≈ 0.2Γ and the symmetric mode linewidths become more superradiant *γ*_s_ ≈ 1.8Γ.

The lineshifts *δω*_*n*_, however, only agree when $kl\gtrsim \pi /2$. As the separation becomes small $kl\lesssim \pi /2$, the line shift of the point electric dipole model begins to separate from the corresponding line shift of the finite-size resonator model. The line shift of the finite-size resonator model here is ${{\rm{\Omega }}}_{{\rm{a}}}^{(1)}-{\omega }_{0}=-({{\rm{\Omega }}}_{{\rm{s}}}^{(1)}-{\omega }_{0})\simeq 2.5{\rm{\Gamma }}$. For $kl\lesssim \pi /2$, the antisymmetric mode line shift of the point electric dipole model is blue shifted from Ω_0_, and the symmetric mode red shifted.

In Fig. 2, the nanorods have length ${H}_{0}=0.24{\lambda }_{0}$ and radius *a* = *λ*_p_/5. In the Supplementary Material, we show the line shifts and linewidths when driven on resonance for longer and shorter rods, still with radius *a* = *λ*_p_/5. The longer rods have length ${H}_{l}=2{H}_{0}\simeq 0.27{\lambda }_{l}$, where ${\lambda }_{l}\simeq 1540\,{\rm{nm}}$ is the resonance wavelength of the longer rod, and shorter rods ${H}_{t}={H}_{0}/2\simeq 0.18{\lambda }_{t}$, where ${\lambda }_{t}\simeq 570\,{\rm{nm}}$. For both longer and shorter rod systems, the point dipole approximation becomes valid when the separation is $kl\gtrsim \pi /2$, where *k* is the resonance wavenumber of the light. When we make small changes to the nanorod radius *λ*_p_/6 < *a* < *λ*_p_/4; we also find the point dipole approximation is valid for separations $kl\gtrsim \pi /2$.

### Two interacting pairs of nanorods

In this section, we analyze two parallel pairs of horizontal nanorods. Firstly, we treat the interacting pairs as four discrete finite-size nanorods that have a non-vanishing polarization density. Secondly, we optimize the model by treating the two pairs as two effective metamolecules. In each case, we compare the model to four discrete point electric dipoles.

#### Four discrete nanorods

**Figure 3:**
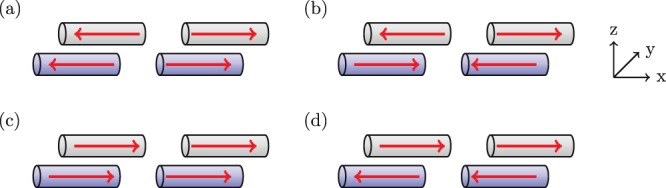
The eigenmodes of two horizontal pairs of nanorods (electric dipoles) shown schematically. The red arrows indicate the phase of current oscillations, the shading indicates nanorods in a shared plane. The nanorods are located at ${{\bf{r}}}_{\pm ,1}=[{\lambda }_{0}/2,\pm \,l/2,0]$ and ${{\bf{r}}}_{\pm ,2}=[-{\lambda }_{0}/2,\pm \,l/2,0]$, where ${\lambda }_{0}$ is the resonance wavelength of a single rod. The modes are designated as follows: (**a**) E1a; (**b**) E2a; (**c**) E1s; and (**d**) E2s.

In general, we position the *j*th pair of nanorods at **r**_±,*j*_ = [*x*_*j*_, *y*_*j*_ ± *l*/2, *z*_*j*_]. In our example, we set *y*_*j*_ = *z*_*j*_ = 0, *kx*_*j*_ = *π* for all *j*, and vary the parameter *l*. When the interactions between individual nanorods are considered, there are four collective modes, see Fig. 3. When each parallel pair of nanorods are symmetrically excited, they can be approximated as out-of-phase (E1a) and in-phase (E1s) effective electric dipoles, see Fig. 3(a,c), respectively. When the nanorods in each pair are antisymmetrically excited, they can be likened to two resonators with both electric quadrupole and magnetic dipole moments, where each pair are out-of-phase (E2a) or in-phase (E2s), see Fig. 3(b,d), respectively.

**Figure 4:**
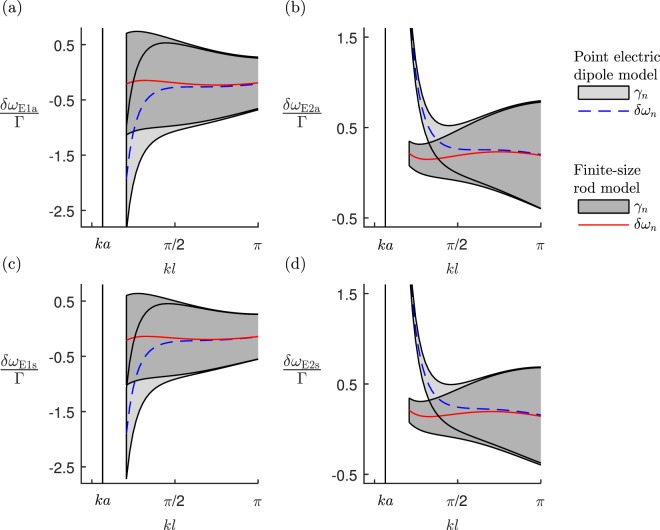
The radiative resonance line shifts *δω* = −(Ω_*n*_ − ω_0_) and linewidths *γ*_*n*_, of two pairs of horizontal nanorods and two pairs of point electric dipoles located at ${{\bf{r}}}_{\pm ,1}=[{\lambda }_{0}/2,\pm \,l/2,0]$ and ${{\bf{r}}}_{\pm ,2}=[-{\lambda }_{0}/2,\pm \,l/2,0]$, as a function of the separation *l,* see Fig. 3. We show the collective modes: (**a**) E1a; (**b**) E2a; (**c**) E1s; and (**d**) E2s. For the nanorod parameters and plot descriptions see Fig. 2 caption.

In Fig. 4, we show the collective mode resonance line shifts and linewidths of four point electric dipole resonators and those of four interacting finite-size nanorods. Again, we find the collective mode decay rates of the different models qualitatively agree throughout the range of *kl*; while the line shifts only agree when $kl\gtrsim \pi /2$. The E1a and E1s modes behave very similarly, as do the E2a and E2s modes. The deviation of the point electric dipole approximation’s line shift from the nanorod model’s, begins to occur when the separation between parallel pairs of rods is $kl\simeq \pi /2$. Here, both the E1a and E1s modes are superradiant with ${\gamma }_{{\rm{E1a}}}\simeq 1.6{\rm{\Gamma }}$ and ${\gamma }_{{\rm{E1a}}}\simeq 1.4{\rm{\Gamma }}$. Conversely, here, the E2a and E2s modes are subradiant with ${\gamma }_{{\rm{E2a}}}\simeq {\gamma }_{{\rm{E2s}}}\simeq 0.6{\rm{\Gamma }}$.

At $kl\simeq 9\pi /10$, the E1s mode becomes noticeably subradiant with ${\gamma }_{{\rm{E1s}}}\simeq 0.9{\rm{\Gamma }}$. Here also, the E2a mode becomes noticeably superradiant with ${\gamma }_{{\rm{E2a}}}\simeq 1.1{\rm{\Gamma }}$. The E1a mode remains superradiant while the E2s mode remains subradiant throughout the range.

#### Two effective metamolecules

When we approximate each pair of nanorods as an effective single resonator; if the nanorods’ current oscillations are in-phase there is an effective electric dipole resonator, if the current oscillations are out-of-phase there is an effective resonator with both electric quadrupole and magnetic dipole responses^35^. In principle, cross coupling can occur between the effective resonators whereby an in-phase pair and an out-of-phase pair get mixed due to interactions. However, here we consider only two interacting in-phase pairs, and separately, two out-of-phase pairs; neglecting such processes. There are two modes of collective oscillation for each effective resonator system; symmetric and antisymmetric. We also designate these as E1s and E1a, respectively, when the nanorods within each pair are in-phase, and E2s and E2a, respectively, when the nanorods within each pair are out-of-phase.

In Fig. 2, we calculated the collective mode decay rates for two parallel nanorods. These decay rates, *γ*_s_ and *γ*_a_, provide us the total decay rate for in-phase and out-of-phase pairs of nanorods, respectively. Also in Fig. 2, we calculated the line shifts of the collective modes, this allows us to determine the resonance frequencies for in-phase and out-of-phase pairs of nanorods; Ω_s_, and Ω_a_, respectively. In general, Ω_s_, Ω_a_ ≠ *ω*_0_, this means that in our effective resonator model, the diagonal elements of ${\mathscr{C}}$ also contain the imaginary component; $${\rm{Im}}{[\begin{array}{c}{\mathscr{C}}\end{array}]}_{jj}=\delta {\omega }_{{\rm{s}},{\rm{a}}}$$, where *δω*_s,a_ are the line shifts of two in-phase and out-of-phase parallel nanorods, respectively, see Fig. 2.

**Figure 5:**
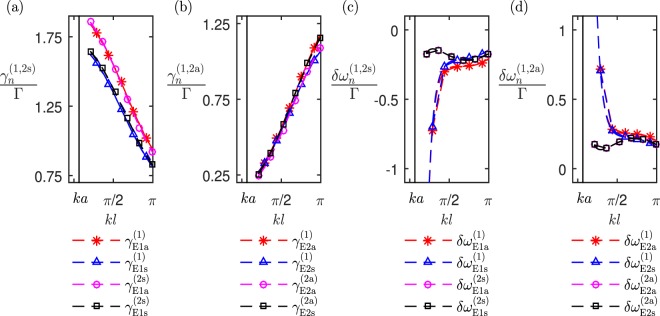
The radiative resonance linewidths ${\gamma }_{n}^{(1,\mathrm{2s},\mathrm{2a})}$ and line shifts $\delta {\omega }_{n}^{(1,\mathrm{2s},\mathrm{2a})}$ for the collective eigenmodes of two horizontal pairs of nanorods and two pairs of point electric dipoles located at ${{\bf{r}}}_{\pm ,1}=[{\lambda }_{0}/2,\pm \,l/2,0]$ and ${{\bf{r}}}_{\pm ,2}=[\,-\,{\lambda }_{0}/2,\pm l/2,0]$ as a function of the parameter *l.* We show: the linewidths ${\gamma }_{n}^{\mathrm{(1)}}$ and line shifts $\delta {\omega }_{n}^{\mathrm{(1)}}$ for the *n* = (E1a, E1s, E2a, E2s) collective modes of the point electric dipole model; the linewidths ${\gamma }_{n}^{(2{\rm{s}})}$ and line shifts $\delta {\omega }_{n}^{(2{\rm{s}})}$ for the *n* = (E1a, E1s) collective modes of the in-phase effective molecules; and the linewidths ${\gamma }^{(2{\rm{a}})}$ and line shifts $\delta {\omega }_{n}^{(2{\rm{a}})}$, for the *n* = (E2a, E2s) out-of-phase effective metamolecules. For the nanorod parameters see Fig. 2 caption.

In Fig. 5, we show how the radiative linewidths $[{\gamma }_{{\rm{E}}1{\rm{a}}}^{(2{\rm{s}})},{\gamma }_{{\rm{E}}1{\rm{s}}}^{(2{\rm{s}})}$, ${\gamma }_{{\rm{E2a}}}^{(\mathrm{2a})}$, ${\gamma }_{{\rm{E2s}}}^{(\mathrm{2a})}]$, and line shifts $[\delta {\omega }_{{\rm{E1a}}}^{(\mathrm{2s})}$, $\delta {\omega }_{{\rm{E1s}}}^{(\mathrm{2s})}$, $\delta {\omega }_{{\rm{E2a}}}^{(\mathrm{2a})}$, $\delta {\omega }_{{\rm{E2s}}}^{(\mathrm{2a})}]$ of the collective modes of oscillation of the *N* = 2 effective interacting pairs of nanorods (denoted by the superscripts (2s) for in-phase and (2a) for out-of-phase pairs) vary with the parameter *l*, and compare to the corresponding modes’ line widths and shifts of *N* = 4 point electric dipole system (denoted by the superscript (1)).

The linewidths resulting from the interacting *N* = 4 point electric dipoles closely agree with those of the *N* = 2 effective metamolecule models, both when in-phase and when out-of-phase. Also, comparing Fig. 5(a,b), with Fig. 4, the effective metamolecules’ linewidths closely match those of the finite-size nanorod model.

The point electric dipole model’s line shifts in Fig. 5(c,d) begin to separate from the corresponding effective metamolecule line shifts when $kl\simeq \pi /2$. As *kl* reduces, the point electric dipole model’s line shifts for the E2a and E2s (E1a and E1s) modes blue shift (red shift) from the out-of-phase (in-phase) effective metamolecule’s corresponding modes. Comparing Fig. 5(c,d) with the corresponding line shifts in Fig. 4, the line shifts of the finite-size nanorod model closely agree with those of the effective metamolecule model.

## Conclusions

Understanding the complex EM interactions in resonator ensembles is important for the design of metamaterials. Our circuit element resonator model provides an efficient way of understanding the dynamics of the system without having to fully solve Maxwell’s equations. We have studied the strong collective modes of current oscillations resulting from the EM interactions in closely spaced resonator systems. These collective modes have an associated radiative response that can be either superradiant or subradiant, and together with the resonance line shift, is strongly influenced by the spatial separation of the resonators. Though in this work, we have considered all the resonance frequencies of the resonators to be equal, variation in the resonances (inhomogeneous broadening) can generally suppress the collective radiation interactions^38^.

We have analyzed, in detail, the validity of the point electric dipole approximation of interacting resonators in small systems; demonstrating how we can model plasmonic nanorod systems both as point electric dipole resonators and accounting for their finite-size and geometry. In particular, we have determined how interacting discrete nanorods with an appreciable range of lengths centered on ${H}_{0}\simeq 0.24{\lambda }_{0}\simeq 210\,{\rm{nm}}$ can be approximated as interacting point electric dipoles, especially when their separation is greater than $kl\simeq \pi /2$. For closely spaced resonators $kl\lesssim \pi /2$, their finite-size and geometry becomes increasingly important.

An alternative approach for treating each resonator as a separate meta-atom is to model closely spaced resonators as a single effective metamolecule, reducing the number of degrees of freedom. In principle, this could be extended to other more complex effective metamolecules, e.g, toroidal metamolecules^32^.

## Supplementary information

Supplementary material to: Point-dipole approximation for small systems of strongly coupled radiating nanorods
