# Supplementary material for: Point-dipole approximation for small systems of strongly coupled radiating nanorods
Source: Sci Rep. 2019 Apr 5;9:5707. doi: 10.1038/s41598-019-41327-6 (PMC6450961; doi:10.1038/s41598-019-41327-6)
Supplement: Supplementary file 1 — Supplementary material to: Point-dipole approximation for small systems of strongly coupled radiating nanorods [file 41598_2019_41327_MOESM1_ESM.pdf]

Supplementary material to: Point-dipole approximation for small systems of strongly coupled radiating nanorods

Derek W. Watson[1*], Stewart D. Jenkins[1], Vassili A. Fedotov[2], and Janne Ruostekoski[1,3]

[1]Mathematical Sciences and Centre for Photonic Metamaterials, University of Southampton, Southampton SO17 1BJ, United Kingdom
[2]Optoelectronics Research Centre and Centre for Photonic Metamaterials, University of Southampton, Southampton SO17 1BJ, United Kingdom
[3]Department of Physics, Lancaster University, Lancaster, LA1 4YB, United Kingdom
[1]*derek.w.watson@gmail.com

Introduction

In the main text, we provided a brief overview of our method. Here, in this Supplementary Material, we provide more detail for the interested reader. The method is based on the formalism introduced in Reference 1.

Electromagnetic Radiated fields

In the general model of circuit resonator interactions with EM fields, we assume that the charge and current sources are initially driven by an incident electric displacement field $\mathbf{D}_{\text{in}}(\mathbf{r},t)$, and magnetic induction $\mathbf{B}_{\text{in}}(\mathbf{r},t)$, with frequency Ω_0. The electric $\mathbf{E}_{\text{sc},j}(\mathbf{r},t)$ and magnetic $\mathbf{H}_{\text{sc},j}(\mathbf{r},t)$ fields scattered by resonator j, are a result of its oscillating polarization $\mathbf{P}_j(\mathbf{r},t)$ and magnetization $\mathbf{M}_j(\mathbf{r},t)$ sources. In general, the electric and magnetic fields are related to the electric displacement and magnetic induction through the auxiliary equations

$$\mathbf{D}(\mathbf{r},t) = \varepsilon_0\mathbf{E}(\mathbf{r},t) + \mathbf{P}(\mathbf{r},t), \quad \mathbf{H}(\mathbf{r},t) = \frac{1}{\mu_0}\mathbf{B}(\mathbf{r},t) - \mathbf{M}(\mathbf{r},t), \tag{1}$$

where ε_0 and μ_0 are the free-space permittivity and permeability, respectively. When analyzing the EM fields and resonators, we adopt the rotating wave approximation where the dynamics is dominated by Ω_0. In this work, all the EM field and resonator amplitudes refer to the slowly-varying versions of the positive frequency components of the corresponding variables, where the rapid oscillations $e^{-i\Omega_0 t}$ due to the dominant laser frequency has been factored out. The scattered electric and magnetic fields are then given by[1]

$$\mathbf{E}_{\text{sc},j}(\mathbf{r},t) = \frac{k^3}{4\pi\varepsilon_0}\left[\int d^3r'\,\mathbf{G}(\mathbf{r}-\mathbf{r}')\cdot\mathbf{P}_j(\mathbf{r}',t) + \frac{1}{c}\mathbf{G}_\times(\mathbf{r}-\mathbf{r}')\cdot\mathbf{M}_j(\mathbf{r}',t)\right], \tag{2}$$

$$\mathbf{H}_{\text{sc},j}(\mathbf{r},t) = \frac{k^3}{4\pi}\left[\int d^3r'\,\mathbf{G}(\mathbf{r}-\mathbf{r}')\cdot\mathbf{M}_j(\mathbf{r}',t) - c\mathbf{G}_\times(\mathbf{r}-\mathbf{r}')\cdot\mathbf{P}_j(\mathbf{r}',t)\right], \tag{3}$$

where $k = \Omega/c$. Explicit expression for the radiation kernels are[1]

$$\mathbf{G}(\mathbf{r}) = i\left[\frac{2}{3}\mathbf{I}h_0^{(1)}(kr) + \left(\frac{\mathbf{rr}}{r^2} - \frac{\mathbf{I}}{3}\right)h_2^{(1)}(kr)\right] - \frac{4\pi}{3}\mathbf{I}\delta(k\mathbf{r}), \quad \mathbf{G}_\times(\mathbf{r}) = \frac{1}{k}\nabla\times\frac{e^{ikr}}{kr}\mathbf{I}, \tag{4}$$

Here: the dyadic $\mathbf{rr}$, is the outer product of $\mathbf{r}$ with itself; $\mathbf{I}$ is the identity matrix; and $h_n^{(1)}(x)$ are spherical Hankel functions of the first kind, of order n, defined by

$$h_0^{(1)}(x) = -i\frac{e^{ix}}{x}, \quad h_2^{(1)}(x) = i\left[\frac{1}{x} + i\frac{3}{x^2} - \frac{3}{x^3}\right]e^{ix}. \tag{5}$$

The radiation kernel $\mathbf{G}(\mathbf{r}-\mathbf{r}')$ determines the electric (magnetic) field at $\mathbf{r}$, from a polarization (magnetization) source at $\mathbf{r}'$[2]. The cross kernel $\mathbf{G}_\times(\mathbf{r}-\mathbf{r}')$ determines the electric (magnetic) field at $\mathbf{r}$, from a magnetization (polarization) source at $\mathbf{r}'$. In general, for sources other than point resonators, the scattered field equations are not readily solved for $\mathbf{P}_j(\mathbf{r},t)$ and $\mathbf{M}_j(\mathbf{r},t)$. When resonators are separated by distances less than, or of the order of a wavelength, a strongly coupled system results.

The total electric $\mathbf{E}_{\text{ext},j}(\mathbf{r},t)$ and magnetic $\mathbf{H}_{\text{ext},j}(\mathbf{r},t)$ fields external to resonator j comprise the incident fields and those fields scattered from all other resonators,

$$\mathbf{E}_{\text{ext},j}(\mathbf{r},t) = \frac{1}{\varepsilon_0}\mathbf{D}_{\text{in}}(\mathbf{r},t) + \sum_{i\neq j}\mathbf{E}_{\text{sc},i}(\mathbf{r},t)\,, \quad \mathbf{H}_{\text{ext},j}(\mathbf{r},t) = \frac{1}{\mu_0}\mathbf{B}_{\text{in}}(\mathbf{r},t) + \sum_{i\neq j}\mathbf{H}_{\text{sc},i}(\mathbf{r},t)\,. \tag{6}$$

The total driving of the charge and current oscillations within the resonator is provided by the external electric and magnetic fields, Equations (6), aligned along the direction of the source, providing a net electromagnetic force (emf) and flux[1], $\mathscr{E}_{\text{ext},j}$ and $\Phi_{\text{ext},j}$, respectively. We define the external emf and flux as[1]

$$\mathscr{E}_{\text{ext},j} = \frac{1}{\sqrt{\omega_j L_j}}\int d^3r\,\frac{\mathbf{P}_j(\mathbf{r},t)}{Q_j}\cdot\mathbf{E}_{\text{ext},j}(\mathbf{r},t)\,, \quad \Phi_{\text{ext},j} = \frac{1}{\sqrt{\omega_j L_j}}\int d^3r\,\frac{\mathbf{M}_j(\mathbf{r},t)}{I_j}\cdot\mathbf{H}_{\text{ext},j}(\mathbf{r},t)\,, \tag{7}$$

where Q_j has units of charge and $I_j = \dot{Q}_j$, is the rate of change of Q_j. The external emf and flux can be decomposed into contributions from the incident ($\mathscr{E}_{\text{in},j}$ and $\Phi_{\text{in},j}$) and scattered ($\mathscr{E}^{\text{sc}}_{i,j}$ and $\Phi^{\text{sc}}_{i,j}$) fields,

$$\mathscr{E}_{\text{ext},j} = \mathscr{E}_{\text{in},j} + \sum_{i\neq j}\mathscr{E}^{\text{sc}}_{i,j}\,, \quad \Phi_{\text{ext},j} = \Phi_{\text{in},j} + \sum_{i\neq j}\Phi^{\text{sc}}_{i,j}\,. \tag{8}$$

The driving by the incident displacement field and magnetic induction is $\mathscr{E}_{\text{in},j}$ and $\Phi_{\text{in},j}$, respectively. The driving of resonator j by the scattered electric and magnetic fields from resonator i is the emf $\mathscr{E}^{\text{sc}}_{i,j}$ and flux $\Phi^{\text{sc}}_{i,j}$, respectively. The total driving of a resonator can be summarized by the external driving $F_{\text{ext},j}$, the sum of the incident $F_{\text{in},j}$ and scattered $F_{\text{sc},j}$ driving contributions, respectively, where[1]

$$F_{\text{ext},j} = F_{\text{in},j} + F_{\text{sc},j} = F_{\text{in},j} + \sum_{i\neq j}\mathscr{C}_{ij}\,, \tag{9}$$

where the components

$$F_{\text{in},j} = \frac{i}{\sqrt{2}}\left[\mathscr{E}_{\text{in},j} + i\omega_j\Phi_{\text{in},j}\right], \quad [\mathscr{C}]_{i\neq j} = \frac{i}{\sqrt{2}}\left[\mathscr{E}^{\text{sc}}_{i,j} + i\omega_j\Phi^{\text{sc}}_{i,j}\right]. \tag{10}$$

Normal modes

In order to express the coupled equations for the EM fields and resonators, in the main text we introduced the slowly varying normal mode oscillator amplitudes[1] $b_j(t)$,

$$b_j(t) = \frac{1}{\sqrt{2\omega_j}}\left[\frac{Q_j(t)}{\sqrt{C_j}} + i\frac{\phi_j(t)}{\sqrt{L_j}}\right], \tag{11}$$

with generalized coordinate the charge $Q_j(t)$ and $\phi_j(t)$ its conjugate momentum.

The normal mode oscillators $b_j(t)$ represent the current oscillations of the resonator. These current oscillations are subject to radiative damping due to their own emitted radiation. The Hamiltonian equation of motion for Q and ϕ [$\dot{Q} = I$; and $\dot{\phi} = \mathscr{E}$] together with the scattered EM fields from all the resonators results in a linear system of equations for $b_j(t)$. For a system which comprises N resonators, we write the system as[1]

$$\dot{\mathbf{b}} = \mathscr{C}\mathbf{b} + \mathbf{F}_{\text{in}}\,, \tag{12}$$

where $\mathbf{b}$ is a column vector of N normal oscillator amplitudes

$$\mathbf{b} = \begin{bmatrix} b_1 \\ b_2 \\ \vdots \\ b_N \end{bmatrix}, \tag{13}$$

$\mathbf{F}_{\text{in}}$ is a column vector formed by the first equation in Equation (10). The matrix $\mathscr{C}$ describes the interactions between the resonator's self-generated EM fields (diagonal elements) and those scattered from different resonators [off-diagonal elements; the interaction terms in, the second equation of, Equation (10)][1]. The diagonal elements of $\mathscr{C}$ contain the resonators' total decay rate Γ_j and resonance frequency shift[1],

$$[\mathscr{C}]_{j,j} = -\frac{\Gamma_j}{2} - i(\omega_j - \Omega_0)\,. \tag{14}$$

Although generally the emitters can have different resonance frequencies, here, for simplicity, we focus on the case of equal frequencies, i.e., $\omega_j = \omega_0$, for all j.

Collective eigenmodes

Strong multiple scattering results in collective excitation modes of the system. The collective modes of current oscillation within the system are described by the eigenvectors $\mathbf{v}_n$ of the interaction matrix $\mathscr{C}$. The decay rate and resonance frequency shift of the mode are described by the real and imaginary parts of the corresponding eigenvalues ξ_n,

$$\xi_n = -\frac{\gamma_n}{2} - i(\Omega_n - \Omega_0)\,. \tag{15}$$

The number of collective modes is determined by the number of resonators N. The collective eigenmodes can then exhibit different resonance frequencies and linewidths and line shifts[1]. The different modes' emitted radiation may be suppresed by the metameterial (subradiant with $\gamma_n < \Gamma$) or enhanced (superradiant with $\gamma_n > \Gamma$).

Point multipole approximation

The general model of interacting resonators formally introduced in Reference 1, and summarized above, can be applied to any type of circuit element resonator. In practice, however, one tends to apply approximations to the intrinsic structure of the resonator. One approach, particularly when the size of the resonator is less than the incident wavelength, is to treat the resonator as a point multipole source[3–5]. Split ring resonators are dominated by electric and magnetic dipole radiation, and a point dipole approximation has been used successfully to study the collective effects in planar resonator arrays, e.g., the transmission properties[6,7] and the development of an electron-beam-driven light source from the collective response[8].

The point dipole approximation alone can not always accurately model interacting resonators. It may be impractical to treat the constituent dipoles separately and higher order multipoles may be more appropriate. In Reference 9, the point multipole approximation was extended to include the point electric quadrupole. In principle, the model can be extended to include any arbitrary multipole. Here, we approximate our resonators as nanorods (cylinders) with radius a and height H. Each nanorod comprises a finite distribution of atomic point electric dipoles along the axis of the rod. Hence, $\mathbf{M}_j(\mathbf{r},t) \simeq 0$, and we only consider the point electric dipole.

Point electric dipole approximation

In the point electric dipole approximation, the polarization density is given by[1] (see also the main text),

$$\mathbf{P}_j(\mathbf{r},t) = Q_j H_j \hat{\mathbf{d}}_j \delta(\mathbf{r}-\mathbf{r}_j)\,, \tag{16}$$

where H_j has units of length and $\hat{\mathbf{d}}_j$ is the orientation vector of the dipole. The electric field follows from inserting Equation (16) into Equation (2). Similarly, the emf resulting from the interaction of dipoles $i \neq j$ is[1]

$$\mathscr{E}^{\mathrm{sc}}_{i,j} = \sqrt{\Gamma_{\mathrm{E1},i}\Gamma_{\mathrm{E1},j}}\frac{3}{2}\hat{\mathbf{d}}_i \cdot \mathbf{G}(\mathbf{r}_i - \mathbf{r}_j) \cdot \hat{\mathbf{d}}_j \frac{b_j}{\sqrt{2}}\,. \tag{17}$$

Here, $\Gamma_{\mathrm{E1},j}$ is the radiative emission rate of the dipole[1],

$$\Gamma_{\mathrm{E1},j} = \frac{C_j H_j^2 \omega^4}{6\pi\varepsilon_0 c^3}\,. \tag{18}$$

The total decay rate Γ_j of the point electric dipole comprises the radiative emission rate $\Gamma_{\mathrm{E1},j}$ and ohmic losses $\Gamma_{\mathrm{O},j}$;

$$\Gamma_j = \Gamma_{\mathrm{E1},j} + \Gamma_{\mathrm{O},j}\,. \tag{19}$$

Finite size nanorod model

For a nanorod aligned along the z-axis, the polarization density is

$$\mathbf{P}_j(\mathbf{r},t) = \frac{Q_j}{\pi a^2}\hat{\mathbf{z}}\Theta(a-\rho)\Theta(H_j/2 - z)\Theta(H_j/2 + z)\,, \tag{20}$$

where Θ is the Heaviside function and $\rho < a$. The electric field $\mathbf{E}_{\mathrm{sc},j}$ is obtained by inserting Equation (20) into Equation (2) and integrating over the volume of the nanorod. The emf resulting from nanorods i and j interacting is

$$\mathscr{E}^{\mathrm{sc}}_{i,j} = \frac{k^3}{4\pi\varepsilon_0 Q_i \sqrt{\omega_j L_j}} \int \mathrm{d}^3 r\, \mathrm{d}^3 r'\, \mathbf{P}_i(\mathbf{r},t) \cdot \mathbf{G}(\mathbf{r}_i - \mathbf{r}_j) \cdot \mathbf{P}_j(\mathbf{r}',t)\,. \tag{21}$$

Equation (21) comprises the integral over the volume of the nanorod located at $\mathbf{r}$ and the volume of the nanorod located at $\mathbf{r}'$. Each nanorod also experiences a radiative decay rate given by Equation (18) and an ohmic loss rate. Its total decay rate is also given in Equation (19).

A finite size effective metamolecule

Thus far, we modeled the interactions between individual nanorods and point electric dipole resonators. When there are a large number of resonators, one may look to optimize the model. Because resonators are often arranged about a lattice framework, this can result in rows of parallel resontors. In our optimization method, we treat closely spaced parallel nanorods as a single effective resonator. In a point multipole approximation, this effective resonator has magnetic dipole and electric quadrupole properties[9]. However, when we extend our finite-size nanorod model to include this effective metamolecule; the effective metamolecule has the same properties as its constituent nanorods; that is electric dipole properties.

We consider two parallel nanorods located at $\mathbf{r}_{\pm,j} = [x_j, y_j \pm l, z_j]$; with corresponding polarization densities $\mathbf{P}_{\pm,j}(\mathbf{r},t)$. When l is small, we approximate the pair as a single metamolecule located at $\mathbf{r}_j = [x_j, y_j, z_j]$. The metamolecule may be symmetrically excited, i.e., $\mathbf{P}_{+,j}(\mathbf{r},t) = \mathbf{P}_{-,j}(\mathbf{r},t)$, or it may be antisymmetrically excited, i.e., $\mathbf{P}_{+,j}(\mathbf{r},t) = -\mathbf{P}_{-,j}(\mathbf{r},t)$, where $\mathbf{P}_{+,j}(\mathbf{r},t)$ is defined in Equation (20). When we consider the interactions between these effective metamolecules, we integrate over the volume of the *pair of nanorods* centered at $\mathbf{r}$, and the *pair* centered at $\mathbf{r}'$.

In our model, we assume that a single nanorod and a point electric dipole have the same radiative decay rate, ohmic loss rate and resonance frequency ω_0 . However, our symmetrically excited or antisymmetrically excited effective metamolecules have radiative decay rates and resonance frequencies different to a single nanorod. We denote the decay rates of symmetrically and antisymmetrically excited nanorods as γ_s and γ_a, respectively, and their resonance frequencies as Ω_s and Ω_a, respectively. In general, $\Omega_\mathrm{s} \neq \Omega_\mathrm{a} \neq \omega_0$. We calculate these decay rates and resonance frequencies in the main text by analyzing a pair of parallel nanorods.

Radiative emission rate of a single nanorod

In the main text we gave the radiative emission rate of a single gold nanorod as $\Gamma_{\mathrm{E1}} \simeq 0.83\Gamma$ with an ohmic loss rate of $\Gamma_\mathrm{O} \simeq 0.17\Gamma$. We also drove our systems at the resonance frequency of the electric dipole ω_0. Here, we provide justification for these parameter choices and calculate ω_0. To do so, we consider the scattering and polarizability of a small metallic nanorod. This allows us to estimate its radiative and ohmic loss rates as well as its resonance frequency.

The analysis relies on the Drude model for the permittivity ε of a metallic resonator

$$\varepsilon(\omega) = \varepsilon_\infty - \frac{\omega_\mathrm{p}^2}{\omega(\omega + i\Gamma_\mathrm{D})}, \tag{22}$$

where: ω is the frequency of the incident light; ε_∞ is the permittivity at infinite frequencies; ω_p is the plasma frequency; and Γ_D is the decay rate of current oscillations within the material.

The scattering cross section σ_{sc} of small particles depends on their polarizability α and frequency of the incident light[10]

$$\sigma_{\mathrm{sc}} = \frac{\omega^4}{6\pi c^4}|\alpha|^2. \tag{23}$$

The polarizability is determined by the particle's physical characteristics, especially its volume V_0 and geometry. The geometry introduces a depolarization effect L on the particle[10]. In the Rayleigh approximation, the polarizability is

$$\alpha_i = V_0 \frac{\varepsilon - 1}{1 + L_i(\varepsilon - 1)}, \quad i = x, y, z. \tag{24}$$

For a cylinder aligned along the z axis, the depolarization factor is[11,12]

$$L_z = 1 - \frac{1}{\sqrt{1+\kappa^2}} \quad \text{and} \quad L_x = L_y = \frac{1}{2\sqrt{1+\kappa^2}}, \tag{25}$$

where $\kappa = 2a/H$ is the aspect ratio of a cylinder with radius a and height H.

The curve produced by the scattering cross section Equation (23) has two Lorentzian profiles with two independent resonance frequencies. The first is associated with the longitudinal polarizability α_z and depolarization factor L_z, the second is associated with the radial polarizability $\alpha_x = \alpha_y$, with depolarization factors $L_x = L_y$.

For gold, the Drude parameters are[13–15]: $\varepsilon_\infty = 9.5$; $\omega_\mathrm{p} \simeq (2\pi)2200$ THz; and $\Gamma_\mathrm{D} \simeq (2\pi)17$ THz. In the Rayleigh approximation (for a cylinder with fixed radius a), the full width at half maximum (FWHM) of σ_{sc} is approximately independent of the cylinder's length and is associated with the ohmic loss rate Γ_O. In Figure 1(a,b), we show Γ_O and resonance frequency ω_0 for a gold nanorod with radius $a = \lambda_\mathrm{p}/5$, where $\lambda_\mathrm{p} = 2\pi c/\omega_\mathrm{p} \simeq 140$ nm is the plasma wavelength. We find $\Gamma_\mathrm{O} \simeq \Gamma_\mathrm{D} \simeq (2\pi)17$ THz.

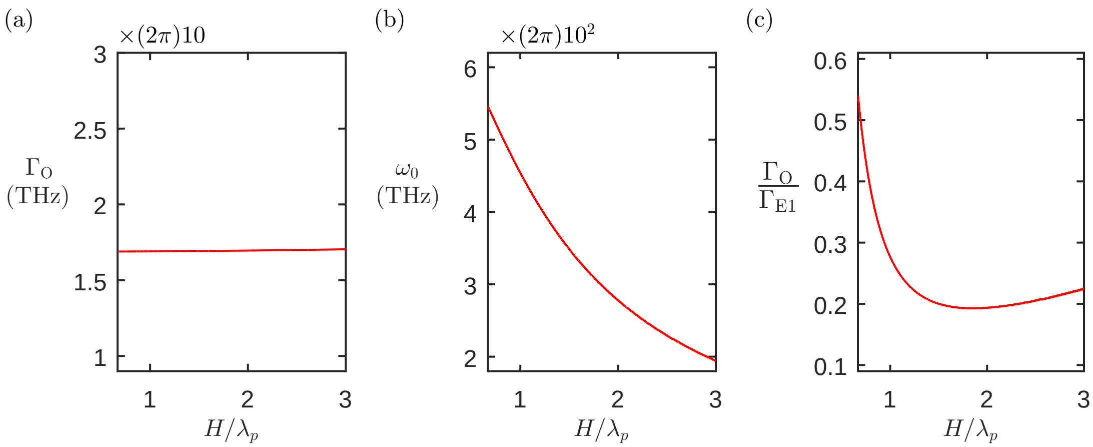

Figure 1. We show: (a) the ohmic loss $\Gamma_{\rm O}$; (b) the resonance frequency ω_0; and (c) the relative radiative decay $\Gamma_{\rm O}/\Gamma_{\rm E1}$, as functions of the nanorod rod length H, for a gold nanorod with radius $a = \lambda_{\rm p}/5$.

For larger particles, the Rayleigh approximation is insufficient and retardation effects must be considered. Mie's formulation accounts for this retardation for spherical particles. For non-spheroidal particles, a generalization of Mie's polarizability was obtained in Reference 16 and successfully used to model the scattering of metallic nanoparticles. The approximate ratio of ohmic losses to radiative decay is

$$\frac{\Gamma_{\rm O}}{\Gamma_{\rm E1}} = -{\rm Im}\left(\frac{3\lambda_0^3}{4\pi^3 a^2 (H(\varepsilon-1)}\right) = \frac{6\Gamma_{\rm D}\omega_{\rm p}^2 c^3}{a^2 H\omega_0^2\left[\omega_0^2\Gamma_{\rm D}^2(\varepsilon_\infty-1)+\left(\omega_0^2(\varepsilon_\infty-1)-\omega_{\rm p}^2\right)^2\right]}. \tag{26}$$

In Figure 1(c), we show the relative decay rates as a function of the nanorod length. When the nanorods are shorter, the ohmic losses are more dominant. For example, when $H = 0.75\lambda_{\rm p}$, the total decay rate Γ is twice that when $H = 1.5\lambda_{\rm p}$, however, the ohmic losses are larger; $\Gamma_{\rm O} \simeq 0.3\Gamma$.

For longer nanorods, $H \simeq 3\lambda_{\rm p}$ the total decay rate reduces. For example, when $H = 3\lambda_{\rm p}$ the total decay rate is approximately one third that when $H = 1.5\lambda_{\rm p}$, however, here the ohmic losses are $\Gamma_{\rm O} \simeq 0.18\Gamma$. In the region $1.25\lambda_{\rm p} \lesssim H \lesssim 1.75\lambda_{\rm p}$, the radiative decay rate is approximately constant, $\Gamma_{\rm E1} \simeq 5\Gamma_{\rm O}$. In the main text, we choose our nanorods to have length $H = 1.5\lambda_p \simeq 0.24\lambda_0$, where $\lambda_0 = 2\pi c/\omega_0 \simeq 860$ nm is the resonance wavelength of this nanorod. This results in a radiative decay rate $\Gamma_{\rm E1} \simeq 0.83\Gamma$ and ohmic loss rate $\Gamma_{\rm O} \simeq 0.17\Gamma$, where Γ is the total decay rate of this nanorod.

The effects of rod length

In the main text, we analyze in detail different systems of gold nanorods with radii $a = \lambda_{\rm p}/5 \simeq 28$ nm and length $H_0 = 1.5\lambda_{\rm p} \simeq 210$ nm. The total decay rate of this nanorod is Γ_0 (we introduce the subscript (0) here for convenience). The corresponding radiative decay and ohmic loss rates are $\Gamma_{\rm E1} \simeq 0.83\Gamma_0$ and $\Gamma_{\rm O} \simeq 0.17\Gamma_0$, respectively. In order to investigate the effect of the rod length on the response, we also consider two separate systems of two interacting symmetric nanorods, still with radii $a = \lambda_{\rm p}/5$. However, in the first system the lengths are significantly shorter, $H_t = H_0/2 \simeq 0.18\lambda_t$, where $\lambda_t \simeq 570$ nm is the resonance wavelength of the short rod, and in the second system, the rod lengths are longer, $H_l = 2H_0 \simeq 0.27\lambda_l$, where $\lambda_l \simeq 1540$ nm is the resonance wavelength of the long rod. For the shorter nanorods, this results in a radiative decay $\Gamma_{\rm E1} \simeq 1.40\Gamma_0$ and ohmic losses $\Gamma_{\rm O} \simeq 0.6\Gamma_0$, where Γ_0 denotes the total decay rate of a nanorod with length H_0. For the longer nanorods, we have $\Gamma_{\rm E1} \simeq 0.29\Gamma_0$ and $\Gamma_{\rm O} \simeq 0.07\Gamma_0$. We compare each pair of nanorods, separately, to two interacting point electric dipoles that possess the same orientation vectors, radiative decay, ohmic losses and resonance frequency as the corresponding nanorods.

We analyze the characteristic response of the system in the absence of driving, studying the characteristic collective modes of current oscillation described by the eigenvectors $\mathbf{v}_n$ with corresponding eigenvectors $\xi_n = -\gamma_n/2 - i(\Omega_n - \omega_0)$ (the real part of which correspond to the collective mode linewidth and the imaginary part the corresponding line shift) of the interaction matrix $\mathscr{C}$.

The two parallel nanorods are located at $\mathbf{r}_1 = -\mathbf{r}_2 = [0, l/2, 0]$. The coupling matrix $\mathscr{C}$ has two eigenmodes of current oscillation: the symmetric mode (denoted by a subscript 's') where the current oscillations are in-phase $\hat{\mathbf{d}}_1 = \hat{\mathbf{d}}_2$; and the antisymmetric mode (denoted by a subscript 'a') where the current oscillations are out-of-phase $\hat{\mathbf{d}}_1 = -\hat{\mathbf{d}}_2$.

In Figure 2, we show the radiative linewidths and line shifts for the collective eigenmodes of two parallel nanorods and two parallel point electric dipoles. In Figure 2(a,b), the nanorods are short ($H = H_0/2$) and in Figure 2(c,d), the nanorods are long

($H = 2H_0$). In both cases, the linewidths γ_n of the collective modes in the point electric dipole approximation qualitatively agree with the corresponding linewidths in our finite size nanorod model. The line shifts $\delta\omega_n$ agree when $kl \gtrsim \pi/2$, where $k = 2\pi/\lambda$ and λ is the resonance wavelength of the nanorod of length H. At $kl \simeq \pi/2$, the line shifts of the point electric dipole approximation show a noticeable deviation from the finite size nanorod model. As the separation become small $kl \lesssim \pi/2$, the point electric dipole model deviates significantly, with the $\delta\omega_a$ blue shifting and $\delta\omega_s$ red shifting from ω, the resonance frequency of the nanorod with length H.

In the main text, we show the linewidths and line shifts for two parallel nanorods with length length H_0. With those, together with Figure 2, we show that our point electric dipole approximation is valid over an appreciable range of rod lengths (centred on H_0) when the relative separation is $kl \gtrsim \pi/2$, where k is the resoannce wavenumber of the light.

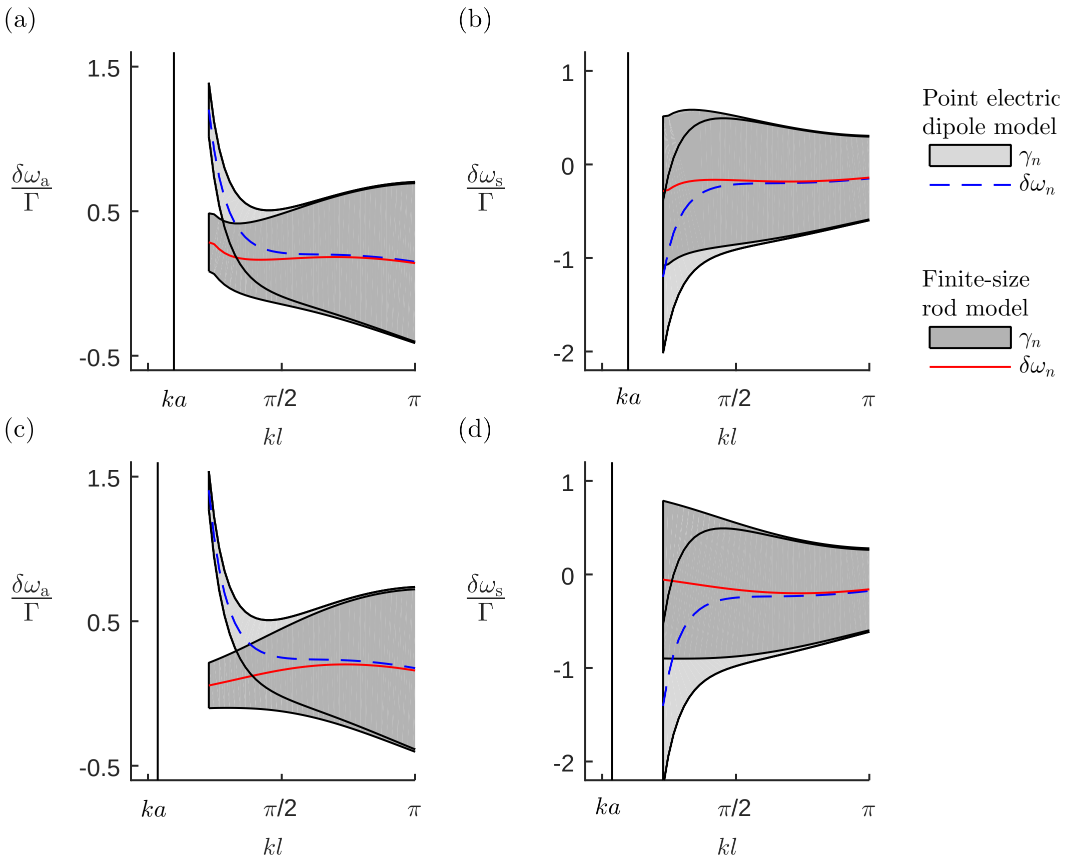

Figure 2. The radiative resonance line shifts $\delta\omega_n = -(\Omega_n - \omega_0)$, and linewidths γ_n, for the collective antisymmetric (a,c) and symmetric (b,d) eigenmodes, as a function of their separation parameter l, for two parallel nanorods. In (a,b), the nanorods have: length $H_t = 0.5H_0 \simeq 0.18\lambda_t$ (where $\lambda_t \simeq 570$ nm is the resonance wavelength of this rod); radiative decay rate $\Gamma_{E1} \simeq 1.4\Gamma_0$; and ohmic loss rate $\Gamma_O \simeq 0.6\Gamma_0$. In (c,d), the nanorods have: length $H_l = 2H_0 \simeq 0.27\lambda_l$ (where $\lambda_l \simeq 1540$ nm is the resonance wavelength of this rod); radiative decay rate $\Gamma_{E1} \simeq 0.29\Gamma_0$; and ohmic loss rate $\Gamma_O \simeq 0.07\Gamma_0$. Each nanorod has radius $a = \lambda_p/5 \simeq 0.032\lambda_0$, where $\lambda_0 \simeq 860$ nm is the resonance wavelength of our reference nanorod with length $H_0 = 1.5\lambda_p \simeq 0.24\lambda_0$ and Γ_0 is is the total decay rate of this nanorod.

References

1. Jenkins, S. D. & Ruostekoski, J. Theoretical formalism for collective electromagnetic response of discrete metamaterial systems. *Phys. Rev. B* **86**, 085116 (2012).
2. Jackson, J. D. *Classical Electrodynamics* (Wiley, New York, 1999).
3. Petschulat, J. *et al.* Simple and versatile analytical approach for planar metamaterials. *Phys. Rev. B* **82**, 075102 (2010).
4. Jenkins, S. D., Ruostekoski, J., Papasimakis, N., Savo, S. & Zheludev, N. I. Many-body subradiant excitations in metamaterial arrays: Experiment and theory. *Phys. Rev. Lett.* **119**, 053901 (2017).
5. Bowen, P. T., Baron, A. & Smith, D. R. Effective-medium description of a metasurface composed of a periodic array of nanoantennas coupled to a metallic film. *Phys. Rev. A* **95**, 033822 (2017).
6. Jenkins, S. D. & Ruostekoski, J. Cooperative resonance linewidth narrowing in a planar metamaterial. *New J. Phys.* **14**, 103003 (2012).
7. Jenkins, S. D. & Ruostekoski, J. Metamaterial transparency induced by cooperative electromagnetic interactions. *Phys. Rev. Lett.* **111**, 147401 (2013).
8. Adamo, G. *et al.* Electron-beam-driven collective-mode metamaterial light source. *Phys. Rev. Lett.* **109**, 217401 (2012).
9. Watson, D. W., Jenkins, S. D. & Ruostekoski, J. Point dipole and quadrupole scattering approximation to collectively responding resonator systems. *Phys. Rev. B* **96**, 035403 (2017).
10. Bohren, C. & Huffman, D. *Absorption and Scattering of Light by Small Particles* (Wiley VCH Verlag GmbH & Co. KGaA, Weinheim, Germany, 2004).
11. Emel'yanov, V. I. Defect-deformational surface roughening and melting and giant enhancement of optical processes at surface of solids. *Laser Phys.* **8**, 937–940 (1998).
12. Emel'yanov, V. I., Zemskov, E. M. & Seminogov, V. N. The influence of collective effects on the local field resonance in the interaction between radiation and a rough solid surface. *Phys. Chem. Mech. Surfaces* **3**, 381–393 (1985).
13. Johnson, P. B. & Christy, R. W. Optical constants of the noble metals. *Phys. Rev. B* **6**, 4370–4379 (1972).
14. Zeman, E. J. & Schatz, G. C. An accurate electromagnetic theory study of surface enhancement factors for silver, gold, copper, lithium, sodium, aluminum, gallium, indium, zinc, and cadmium. *J. Phys. Chem.* **91**, 634–643 (1987).
15. Grady, N., Halas, N. & Nordlander, P. Influence of dielectric function properties on the optical response of plasmon resonant metallic nanoparticles. *Chem. Phys. Lett.* **399**, 167 – 171 (2004).
16. Kuwata, H., Tamaru, H., Esumi, K. & Miyano, K. Resonant light scattering from metal nanoparticles: Practical analysis beyond rayleigh approximation. *Appl. Phys. Lett.* **83**, 4625–4627 (2003).
